# Boceprevir-Based Triple Antiviral Therapy for Chronic Hepatitis C Virus Infection in Kidney-Transplant Candidates

**DOI:** 10.1155/2015/159795

**Published:** 2015-07-16

**Authors:** Mireille Mehawej, Lionel Rostaing, Laurent Alric, Arnaud Del Bello, Jacques Izopet, Nassim Kamar

**Affiliations:** ^1^Faculty of Medical Science, Lebanese University, Hadath, Lebanon; ^2^Department of Nephrology and Organ Transplantation, CHU Rangueil, 31059 Toulouse, France; ^3^INSERM U1043, IFR-BMT, CHU Purpan, 31059 Toulouse, France; ^4^Université Paul Sabatier, 31000 Toulouse, France; ^5^Service de Médecine Interne-Pole Digestif, CHU Purpan, 31059 Toulouse, France; ^6^Laboratoire EA 2405, INSERM IFR31, 31059 Toulouse, France; ^7^Laboratory of Virology, CHU Purpan, 31059 Toulouse, France

## Abstract

*Background*. There are few data on the combination of (pegylated-) interferon- (Peg-IFN-) *α*, ribavirin, and first-generation direct-acting antiviral agents (DAAs). Our aim was to describe the efficacy and safety of Peg-IFN-*α*, ribavirin, and boceprevir in hemodialysis patients. *Patients*. Six hemodialysis patients, chronically infected by genotype-1 HCV, were given Peg-IFN-*α* (135 *µ*g/week), ribavirin (200 mg/d), and boceprevir (2400 mg/d) for 48 weeks. *Results*. At initiation of antiviral therapy, median viral concentration was 5.68 (3.78–6.55) log IU/mL. HCV RNA was undetectable in four of the six patients at week 4 and in all patients at week 24. A breakthrough was observed in two patients between weeks 24 and 48, and a third patient stopped antiviral therapy between weeks 24 and 48 because of severe peripheral neuropathy. At week 48, HCV RNA was undetectable in three patients. Of these, two patients relapsed within a month after antiviral therapy was stopped. Hence, only one patient had a sustained virological response; he was a previous partial responder. Overall, anemia was the main side effect. *Conclusion*. A triple antiviral therapy based on Peg-IFN-*α*, ribavirin, and boceprevir is not optimal at treating hemodialysis patients with chronic HCV infection. Studies using new-generation drugs are required in this setting.

## 1. Introduction

Chronic hepatitis C virus (HCV) infection has a detrimental effect on the health of persons with chronic kidney disease [1]. It leads to higher mortality in maintenance hemodialysis patients compared to noninfected patients and reduces the survival rates of patients undergoing kidney transplantation as well as their grafts [2]. It renders patients with a higher risk of developing diabetes mellitus,* de novo* or recurrent membranoproliferative glomerulonephritis, lymphoproliferative disorders, and fibrosing cholestatic hepatitis after kidney transplantation [3].

Until recently, in the absence of any efficient treatment for HCV infection after kidney transplantation, treating all anti-HCV-positive RNA-positive patients that were candidates for kidney transplantation has been recommended [4].

For several years, standard reduced interferon- (IFN-) alpha or pegylated- (Peg-) IFN-*α*-2a, given as a monotherapy, has been the main treatment given to hemodialysis patients with HCV replication [4]. Because ribavirin has been found to be responsible for hemolytic anemia in patients with impaired kidney function [5] as well as in hemodialysis patients, it was first prohibited and then used at markedly reduced daily doses with careful monitoring for anemia and other adverse effects [1]. HCV-positive dialysis patients that received standard IFN-*α* or Peg-IFN-*α* achieved ≈50% efficacy [6].

First-generation direct-acting antiviral agents (DAAs), such as the protease inhibitors telaprevir and boceprevir, have been developed over the past few years. They have been introduced as an adjunctive therapy for patients with chronic HCV but normal kidney function and have significantly improved the SVR [7]. However, scarce data regarding their use in hemodialysis patients have been published [8–10]. In addition, no data regarding the use of new-generation DAAs, such as sofosbuvir, daclatasvir, simeprevir, or ledipasvir, have been reported in this setting.

The aim of this study was to assess the efficacy and safety of a combined therapy of Peg-IFN, ribavirin, and boceprevir to treat anti-HCV-positive RNA-positive hemodialysis patients who were candidates for kidney transplantation.

## 2. Patients and Methods

Between February 2013 and September 2014, six patients who were chronically infected by genotype-1 HCV and were receiving hemodialysis three times weekly, and who were candidates for kidney transplantation, were treated with Peg-IFN, ribavirin, and boceprevir as a triple therapy, after having given their written informed consent. The study was approved by Toulouse University's IRB. The patients' characteristics are presented in Table 1. There were three men and three women, ranging in age from 39 to 72 years (median: 49.5). Four were candidates for a second or third transplantation. Five of the six patients had previously received interferon for HCV infection: one had a relapse and the other four patients were partial responders.

Peg-IFN was given at the dose of 135 *μ*g/week. Ribavirin was given at the dose of 200 mg/d, three times a week, after each dialysis session. Boceprevir was given at the dose of 800 mg t.i.d. There was no lead-in therapy phase. Therapy was scheduled to last 48 weeks.

A rapid virological response was defined as undetectable HCV RNA at week 4. A sustained virological response was defined as negative HCV RNA six months after the end of therapy. A virological breakthrough was defined as HCV replication during therapy and after a period of nonviral replication.

Patients were followed up every 15 days during the first month and then at three-month intervals for 6 months after therapy was completed. HCV RNA was assessed using the quantitative COBAS Amplicor HCV monitor assay (limit of detection 15 log IU/mL).

## 3. Results

### 3.1. Virological Response

**Figure 1:**
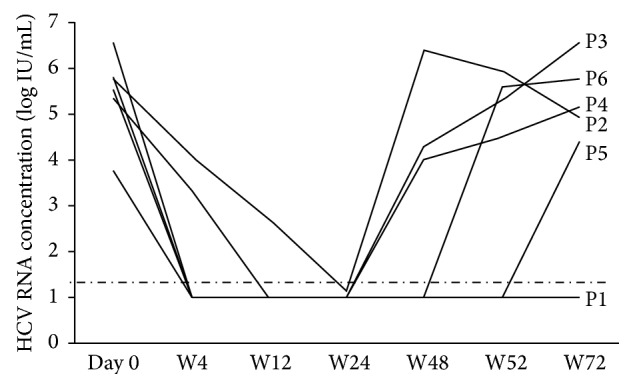
Evolution of hepatitis C virus RNA concentration during and after therapy.

At the initiation of antiviral therapy, median viral concentration was 5.68 (4.3–6.55) log IU/mL. The evolution of HCV concentration is presented in Figure 1. A rapid virological response, that is, undetectable HCV RNA at week 4, was observed in four of the six patients (66.6%). All patients had at least once undetectable HCV RNA during therapy, and HCV RNA was undetectable in all patients at week 24. However, a breakthrough was observed in two patients (P3 and P4) between weeks 24 and 48, and a third patient (P2) had to stop antiviral therapy between weeks 24 and 48 because of severe peripheral neuropathy. At week 48, overall, when therapy was scheduled to finish, HCV RNA was undetectable in three patients. However, of these, two patients relapsed within the month after antiviral therapy had finished. Hence, overall, only one patient (16.7%) had a sustained virological response: he had been a previous partial responder.

### 3.2. Biochemical Response

As is usually observed in hemodialysis patients, liver-enzyme levels were within the normal ranges at the beginning of therapy and also at the end of therapy (data not shown).

### 3.3. Modifications and Tolerance to Antiviral Therapy

Because of fatigue and a flu-like syndrome, Peg-IFN doses were decreased at week 4 from 135 to 90 *μ*g/week in three patients (P2, P3, and P4) and then decreased further at week 24 from 90 to 45 *μ*g/week in one patient (P4). One patient (P2) developed peripheral neuropathy that was attributed to the antiviral therapy, which was stopped at week 28 although the patient was nonviremic.

Overall, as expected, the main adverse effect was hematological tolerance, namely, anemia. Median hemoglobin level decreased from 11.5 (range: 10–13) g/dL at the initiation of antiviral therapy to 9.75 (range: 8.5–11.5) g/dL at week 4 and to 9.55 (range: 8.8–10.8) at week 12. It then remained at 9.55 (range: 8.1–10.5) g/dL until week 24, but then it decreased again to 8.75 (range: 8.4–10.7) g/dL by week 48. At the initiation of ribavirin therapy, three patients were given recombinant erythropoietin (rEPO) at the median dose of 12,000 (range: 8,000–15,000) units/week. From week 4 and until the end of the therapy, rEPO was given to all patients at the median doses of 12,000 (8,000–24,000), 12,000 (8,000–24,000), 16,000 (12,000–30,000), and 18,000 (12,000–30,000) units/week at weeks 4, 12, 24, and 48, respectively.

Ribavirin dose was reduced at week 24 to 200 mg/week in one patient (P4) because of very severe anemia, despite receiving high doses of recombinant erythropoietin. Boceprevir doses were reduced at week 24 to 1600 mg/d in patient 4 and to 800 mg/d in patient 2.

## 4. Discussion

Within the last few years, the first-generation DAA NS3-4A protease inhibitors, that is, boceprevir and telaprevir, have been developed and used in combination with peg-interferon and ribavirin to treat patients with chronic HCV infection [11]. Boceprevir- or telaprevir-based anti-HCV therapy was first used in immunocompetent patients that had preserved kidney function. It significantly improved the sustained virological rate in genotype-1 HCV infected patients, mainly for noncirrhotic patients [7].

In hemodialysis patients, the recommended treatment for chronic HCV infection remains standard or pegylated-interferon [4]. As pointed out in a meta-analysis by Fabrizi et al., the SVR response was 39% after standard IFN-*α* therapy and 31% when Peg-IFN-*α* was used [6]. The dropout rates were, respectively, 19% and 27% [6]. In other studies, although ribavirin accumulates in hemodialysis patients and causes severe hemolytic anemia [5], low doses of ribavirin, that is, 200 mg/day or 200 mg every other day, when added to interferon, improved the SVR [1]. The SVR ranged from 17 to 70%, and the dropout rate was up to 50% [1].

Boceprevir- or telaprevir-based anti-HCV therapy has been used in a small number of hemodialysis patients. Dumortier et al. treated four hemodialysis patients with a combined therapy of Peg-IFN-*α*, ribavirin, and telaprevir [8]. Three of their four patients achieved viral clearance at week 12; however, no further outcomes were reported, and the number of SVRs is unknown. Wiegand et al. treated seven hemodialysis patients with Peg-IFN-*α*, ribavirin, and telaprevir [9]: the duration of treatment ranged from 24 to 47 weeks. A SVR was observed in six of their seven patients [9]. Finally, Knapstein et al. successfully treated a hemodialysis patient with Peg-IFN-*α*, ribavirin, and boceprevir for 48 weeks [10].

In the present study, six patients were given Peg-IFN-*α*, ribavirin, and boceprevir for 48 weeks. Antiviral therapy was stopped in one patient because of severe neuropathy, which was attributed to the interferon therapy. Two breakthrough events were observed in two patients, which required doses of antiviral drugs to be reduced because of adverse events. Overall, three patients were cleared of the virus by the end of therapy. However, two of these patients then relapsed one month and six months after treatment was finished. Hence, overall, only one patient had a sustained virological response.

Eight of the 14 hemodialysis patients that received antiviral triple therapy, who had a sufficiently long follow-up, and were included in either a previously published series or in the present study, had a sustained virological response (57%). The poor results observed in our study are probably related to poor tolerance to the antiviral therapy, which required dose reductions. Similar to previous studies, the main side effect observed in our study was anemia, which required the introduction of rEPO, or an increased dose of rEPO plus blood transfusions. Hence, this triple-therapy strategy did not effectively treat our hemodialysis patients with chronic HCV infection.

Very recently, the use of new-generation DAAs, that is, sofosbuvir combined with daclatasvir, simeprevir, or ledipasvir, has been shown to be highly efficient at treating HCV infection in cirrhotic and noncirrhotic immunocompetent patients [12–16], in liver-transplant patients [17–19] and in some kidney-transplant patients [20]. Individually, daclatasvir, simeprevir, and ledipasvir can be eliminated by the liver and so can be given to hemodialysis patients. However, no data regarding the use of sofosbuvir in patients with a glomerular-filtration rate <30 mL/min exist: hence, a combination of daclatasvir, simeprevir, or ledipasvir is still not recommended in this setting. A phase-II multicenter study has assessed the efficacy and safety of the combination of grazoprevir (MK-5172) and elbasvir (MK-8742) given to patients with chronic hepatitis and chronic kidney disease, including hemodialysis patients (ClinicalTrials.gov: NCT02092350).

In summary, a triple antiviral therapy based on Peg-IFN-*α*, ribavirin, and either telaprevir or boceprevir did not optimally treat hemodialysis patients with chronic HCV infection. Studies using new-generation drugs are required in this population as well as for kidney-transplant patients.

## Figures and Tables

**Table 1:** Patients' characteristics and outcomes.

	Patient 1	Patient 2	Patient 3	Patient 4	Patient 5	Patient 6
Age (years)	43	42	72	56	63	39

Gender	Male	Female	Male	Female	Male	Female

Race	Caucasian	Caucasian	Caucasian	Black	Caucasian	Caucasian

Time on dialysis at initiation of antiviral therapy (months)	4	8	9	60	9	5

Previous kidney transplantation	Yes	Yes	No	Yes	No	Yes

Previous antiviral treatment	Partial responder	Partial responder	Partial responder	Partial responder	Naïve	Relapser

HCV genotype	1	1a	1	1b	1b	1b

Initial HCV RNA concentration (log IU/mL)	4.3	5.63	6.55	5.52	5.8	5.36

Liver fibrosis at the initiation of viral therapy	F2	F2	—	F2	F1	F1-F2

Rapid virological response^*∗*^	Yes	No	Yes	Yes	Yes	Yes

Undetectable viremia at the end of therapy	Yes	No	No	No	Yes	Yes

Sustained virological response^*∗∗*^	Yes	No, stopped therapy at week 28	No, breakthrough between weeks 24 and 48	No, breakthrough between weeks 24 and 48	No, relapse at six months after ceasing therapy	No, relapse at one month after ceasing therapy

Decrease in Peg-IFN dose	No	Yes	Yes	Yes	No	No

Decrease in ribavirin dose	No	No	No	Yes	No	No

Decrease in boceprevir dose	No	Yes	No	Yes	No	No

Concomitant therapy	Amlodipine, bisoprolol, furosemide, calcium, and darbepoetin	Amlodipine, valsartan furosemide, calcium, omeprazole, and darbepoetin	Irbesartan, aspirin, atenolol, simvastatin, and calcium	Calcium, aspirin, omeprazole, and levothyroxine	Simvastatin, amlodipine, and valsartan	Calcium, sevelamer carbonate, alfacalcidol, urapidil, and darbepoetin

Long-term outcome	Had received a kidney transplant at 12 months after the end of therapy and was still not viremic at 4 months after transplantation	Still viremic and on a waiting list	Deceased or on a waiting at list 3 months after antiviral therapy was stopped	Still viremic on a waiting list	Had received a kidney transplant at 1 month after HCV relapse from a HCV-positive donor and was still viremic at 6 months after kidney transplantation	Had received a kidney transplant at 8 months after HCV relapse and was still viremic at 5 months after kidney transplantation

HCV: hepatitis C virus.

^*∗*^Undetectable HCV RNA at week 4; ^*∗∗*^undetectable HCV RNA at 24 weeks after antiviral therapy was completed.
